# Unique Gene Expression Profile of the Proliferating *Xenopus* Tadpole Tail Blastema Cells Deciphered by RNA-Sequencing Analysis

**DOI:** 10.1371/journal.pone.0111655

**Published:** 2015-03-16

**Authors:** Hiroshi Tsujioka, Takekazu Kunieda, Yuki Katou, Katsuhiko Shirahige, Takeo Kubo

**Affiliations:** 1 Department of Biological Sciences, Graduate School of Science, The University of Tokyo, 7-3-1, Hongo, Bunkyo-ku, Tokyo, Japan; 2 Institute of Molecular and Cellular Biosciences, The University of Tokyo, 1-1-1, Yayoi, Bunkyo-ku, Tokyo, Japan; UC Irvine, UNITED STATES

## Abstract

Organ regenerative ability depends on the animal species and the developmental stage. The molecular bases for variable organ regenerative ability, however, remain unknown. Previous studies have identified genes preferentially expressed in the blastema tissues in various animals, but transcriptome analysis of the isolated proliferating blastema cells has not yet been reported. In the present study, we used RNA-sequencing analysis to analyze the gene expression profile of isolated proliferating blastema cells of regenerating *Xenopus laevis* tadpole tails. We used flow cytometry to isolate proliferating cells, and non-proliferating blastema cells, from regenerating tadpole tails as well as proliferating tail bud cells from tail bud embryos, the latter two of which were used as control cells, based on their DNA content. Among the 28 candidate genes identified by RNA-sequencing analysis, quantitative reverse transcription-polymerase chain reaction identified 10 genes whose expression was enriched in regenerating tadpole tails compared with non-regenerating tadpole tails or tails from the tail bud embryos. Among them, whole mount *in situ* hybridization revealed that *chromosome segregation 1-like* and *interleukin 11* were expressed in the broad area of the tail blastema, while *brevican*, *lysyl oxidase*, and *keratin 18* were mainly expressed in the notochord bud in regenerating tails. We further combined whole mount *in situ* hybridization with immunohistochemistry for the incorporated 5-bromo-2-deoxyuridine to confirm that *keratin 18* and *interleukin 11* were expressed in the proliferating tail blastema cells. Based on the proposed functions of their homologs in other animal species, these genes might have roles in the extracellular matrix formation in the notochord bud (*brevican* and *lysyl oxidase*), cell proliferation (*chromosome segregation 1-like* and *keratin 18*), and in the maintenance of the differentiation ability of proliferating blastema cells (*interleukin 11*) in regenerating tadpole tails.

## Introduction

Organ regenerative ability varies depending on the animal species and developmental stage [1]. The molecular bases underlying the variable organ regenerative abilities, however, remain largely unknown. Aiming at identifying the genes involved in organ/tissue regeneration, extensive studies have been performed using various animals with high organ regenerative ability [2–7]. Recent studies have performed transcriptome analyses of the regenerating tissues of amputated organ stumps, using frog tails or limbs or axolotl limbs [8–14]. These studies have identified many genes involved in regeneration, such as *secreted frizzled-related protein 2* [8], *mesendoderm nuclear factor* [9], *neuronal nitric oxide synthase* [10], *gremlin* [11], *prostate stem cell antigen* [13], and *patched-2* [14]. Further characterization of the early processes involved in regenerating organ/tissues will provide important insight into the variable regenerative ability.

To analyze the molecules involved in early processes of organ/tissue regeneration, we focused on the proliferating blastema cells in regenerating *Xenopus laevis* tadpole tails. *X*. *laevis* tadpoles possess high tail regenerative ability except during the ‘refractory period’ when this ability is transiently lost [15]. We previously used the differential display method to comprehensively search for genes whose expression differs in amputated tadpole tail stumps between the ‘refractory period’ and the subsequent ‘post-refractory regeneration period’ [16]. We found that distinct immune responses occur in the amputated tadpole tail stumps between these two periods, and that immunosuppressant treatment drastically restores regenerative ability during the refractory period. Various immune-related genes such as *T Cell Receptor*, a T cell marker, begin to be expressed in the whole tadpole body at the onset of the refractory period, whereas the expression of *forkhead box P3* (*foxp3*), a regulatory T cell marker, is more enriched in the amputated tail stumps in the post-refractory regeneration period than in the refractory period. Based on these findings, we proposed that immature autoreactive immune cells attack blastema cells as ‘non-self’, which results in impaired tail regenerative ability during the refractory period, whereas regulatory T cells suppress autoreactive immune cells, which enables regeneration during the post-refractory regeneration period [16]. The postulated ‘autoreactive immune cells’ as well as ‘autoantigen(s)’ expressed by the *X*. *laevis* tadpole tail blastema, however, have not yet been identified.

In the present study, we aimed to clarify the gene expression profile specific to proliferating *X*. *laevis* tadpole tail blastema cells to identify possible ‘autoantigen(s)’ and candidate genes involved in the early processes of tail regeneration. Among the 10 candidate genes identified, *chromosome segregation 1-like* (*cse1l*) and *interleukin 11* were expressed in a broad area of the blastema that comprises proliferating cells, whereas *brevican*, *lysyl oxidase*, and *keratin 18* were mainly expressed in the proliferating notochord bud cells. These genes might have roles in forming the notochord bud extracellular matrix; regulating immune responses, gene expression, and cell proliferation; and maintaining the differentiation ability of proliferating blastema cells.

## Materials and Methods

### Animals

Animals were treated essentially as described previously [17]. Tadpoles in the tail bud stage were obtained by mating wild-type *Xenopus laevis* adults and maintaining their offspring in the laboratory. Niewkoop and Faber stage [18] (St.) 35-39 tail bud stage tadpoles were used. St. 49-53 tadpoles were purchased from a Japanese company (Watanabe Zoushoku).

All of the surgical manipulations, including the tail amputation, were performed after completely anesthetizing the tadpoles with 0.02% MS222 (Sigma-Aldrich, St. Louis, MO) or ice. These experiments were performed in accordance with the recommendations of the Guidelines for Proper Conduct of Animal Experiments of Science Council of Japan. The protocol was approved by the Committee on the Ethics of Animal Experiments of the Graduate School of Science, the University of Tokyo (Permit Number: 19-14 Z 07-08).

### Immunohistochemistry using anti-bromo-2-deoxyuridine antibody

Immunohistochemistry using anti-bromo-2-deoxyuridine antibody was performed essentially as described previously [19]. Proliferating cells were labeled with 5-bromo-2-deoxyuridine (BrdU) by exposing the tadpoles to water containing 1 mg/ml BrdU (Sigma-Aldrich, St. Louis, MO) for 12 h before sampling. Whole bodies (St. 35-39 tadpoles) or tails (St. 49-53 tadpoles) were fixed with Bouin’s fixative and embedded in Paraplast (McCormick Scientific, St. Louis, MO). Sections cut 10 μm-thick were prepared and rehydrated, and the antigen was retrieved by 2N HCl treatment for 30 min. Immunohistochemical detection of BrdU was performed using mouse anti-BrdU (BD Pharmingen, San Jose, CA, cat. 555627) and Alexa Fluor 555 goat anti-mouse IgG (Invitrogen, Carlsbad, CA, cat. A-21424), followed by counterstaining with 10 μg/ml Hoechst 33342 (Lonza Cologne GmbH, Germany).

### Isolation of proliferating blastema and tail bud embryo cells

Regenerating tail tissues (tail blastemas) were removed with a fine surgical knife from regenerating St. 49-53 tadpole tails 3 days after amputation (dpa). Tail buds were removed with a fine surgical knife from St. 35-39 tail bud stage embryos. Cell dissociation was performed as described previously [20] with minor modifications. In brief, tissues were incubated in dissociation solution (100 U/ml DNase I (Roche Diagnostics, Indianapolis, IN), 0.25 mg/ml Liberase TM research grade (Roche Diagnostics) in phosphate-buffered saline) at 28°C for 30 min. Cells were then passed through a 30-μm filter and washed.

Isolation of the proliferating cells was performed as described previously [21] with minor modifications. In brief, Hoechst 33342 was added to a single cell suspension at a final concentration of 10 μg/ml, the suspension was incubated at 28°C for 30 min, and then washed. Propidium iodide (Molecular Probes, Eugene, OR) was added at a final concentration of 1 μg/ml before cell sorting. The samples were then directly subjected to the flow cytometry using FACS Aria (BD Pharmingen).

### RNA-sequencing analysis

Total RNA was extracted from the proliferating tail blastema cells, whose DNA contents were 4X (‘4X-tail blastema’ cell fraction), non-proliferating tail blastema cells, whose DNA contents were 2X (‘2X-tail blastema’ cell fraction), and the proliferating tail bud cells, whose DNA contents were 4X (‘4X-tail bud’ cell fraction) using an RNeasy mini kit (Qiagen, Valencia, CA). The numbers of cells used were 9.7×10^5^ cells (from ∼1200 tadpoles) for the ‘4X-tail blastema’ cell fraction, 4.0×10^6^ cells (from ∼1200 tadpoles) for the ‘2X-tail blastema’ cell fraction, and 7.6×10^4^ cells (from ∼150 embryos) for the ‘4X-tail bud’ cell fraction. cDNA libraries were generated using TruSeq Stranded Total RNA with Ribo-Zero Gold LT Sample Prep Kit (Illumina, San Diego, CA). We then produced a set of approximately 8.2×10^7^ paired end reads (100 bp×2, insert size 160 bp) from each cDNA library using Hiseq 2500 (Illumina). The dataset was deposited in DNA databank of Japan (Accession No. DRP002318). As very large amounts of samples were needed, it was difficult for us to perform the RNA-sequencing analysis with multiple replicates. Therefore, we performed RNA-sequencing with a single set of samples (and thus the statistical analysis was not applicable to our RNA-sequencing analysis). Rather, we used quantitative reverse transcription-PCR (qRT-PCR) to confirm proliferating tail blastema cell-preferential expression of the identified genes with multiple replicates (lots of samples) as described below.

The sequenced reads were mapped to *X*. *laevis* draft genome 7.0 (LAEVIS_7.repeatMasked.fa) (Xenbase [22]) using TopHat [23,24]. To cover the unannotated transcripts, we generated new gene models based on the mapped reads using Cufflinks [24] with a help of reference model, XlaevisJGIv1.3.primaryTrs.gff3 (Xenbase [22]). Expression levels were estimated and compared using Cufflinks and Cuffdiff programs [24].

### Quantitative reverse transcription-PCR

Expression analysis by quantitative reverse transcription polymerase chain reaction (qRT-PCR) was performed essentially as described previously [17], using total RNA prepared from four sets of the distal half of the normal tails of St. 49-53 tadpoles (n∼25), tail blastemas of the regenerating tadpoles of the same stage 3dpa (n∼100), and tail buds of St. 35-39 tadpoles (n∼100). Total RNA was extracted using an RNeasy mini kit (Qiagen) and reverse-transcribed using PrimeScript RT reagent Kit with gDNA Eraser (Perfect Real Time) (TaKaRa, Japan). qRT-PCR was performed using SYBR premix ExTaq II (Tli RNaseH plus) (TaKaRa), and the amount of each transcript was normalized with that of *ef1α*. The list of primers used in qRT-PCR is shown in S1 Table. Statistical significance was assessed by Dunnett’s test with a cut-off of *P* < 0.05.

### cDNA cloning

To determine the putative primary structure of *interleukin 11*, *l1td1-like*, and *cd200like-related*, we isolated cDNA clones for them. Briefly, total RNA was extracted from three days post amputation tail stumps of St. 49-53 tadpoles using TRIzol (Invitrogen). cDNA was synthesized using SuperScript III First-Strand Synthesis System (Invitrogen) after DNase treatment (DNase I, Invitrogen), or RLM-RCE kit (Life Technologies) for 3’ rapid amplification of a cDNA end (RACE) as described previously [16,25]. *De novo* assembly was performed using CLC Genomics Workbench (CLC Bio), following the manufacturer’s protocol. The sequences were deposited in DNA databank of Japan (accession numbers for the nucleotides of *interleukin 11*, *l1td1-like*, and *cd200like-related* are AB933563, AB933564, AB933565).

### Whole mount *in situ* hybridization

Whole mount *in situ* hybridization was performed as described previously [26] with minor modifications: 50 μg/ml proteinase K was used, and the samples were incubated overnight for four nights with hybridization buffer, and bleached with 10% H_2_O_2_ after the chromogenic reaction.

### Double-staining by BrdU-immunohistochemistry and whole mount *in situ* hybridization

BrdU labeling was performed as described above. Double-staining with whole mount *in situ* hybridization was performed as described previously [26] with minor modifications in addition to those described above: mRNA was detected using HNPP Fluorescent Detection Set (Roche Diagnostics), and the samples were mounted in scale A2 [27], followed by imaging using a confocal microscope.

## Results

### Isolation of proliferating tadpole tail blastema cells

**Fig 1:**
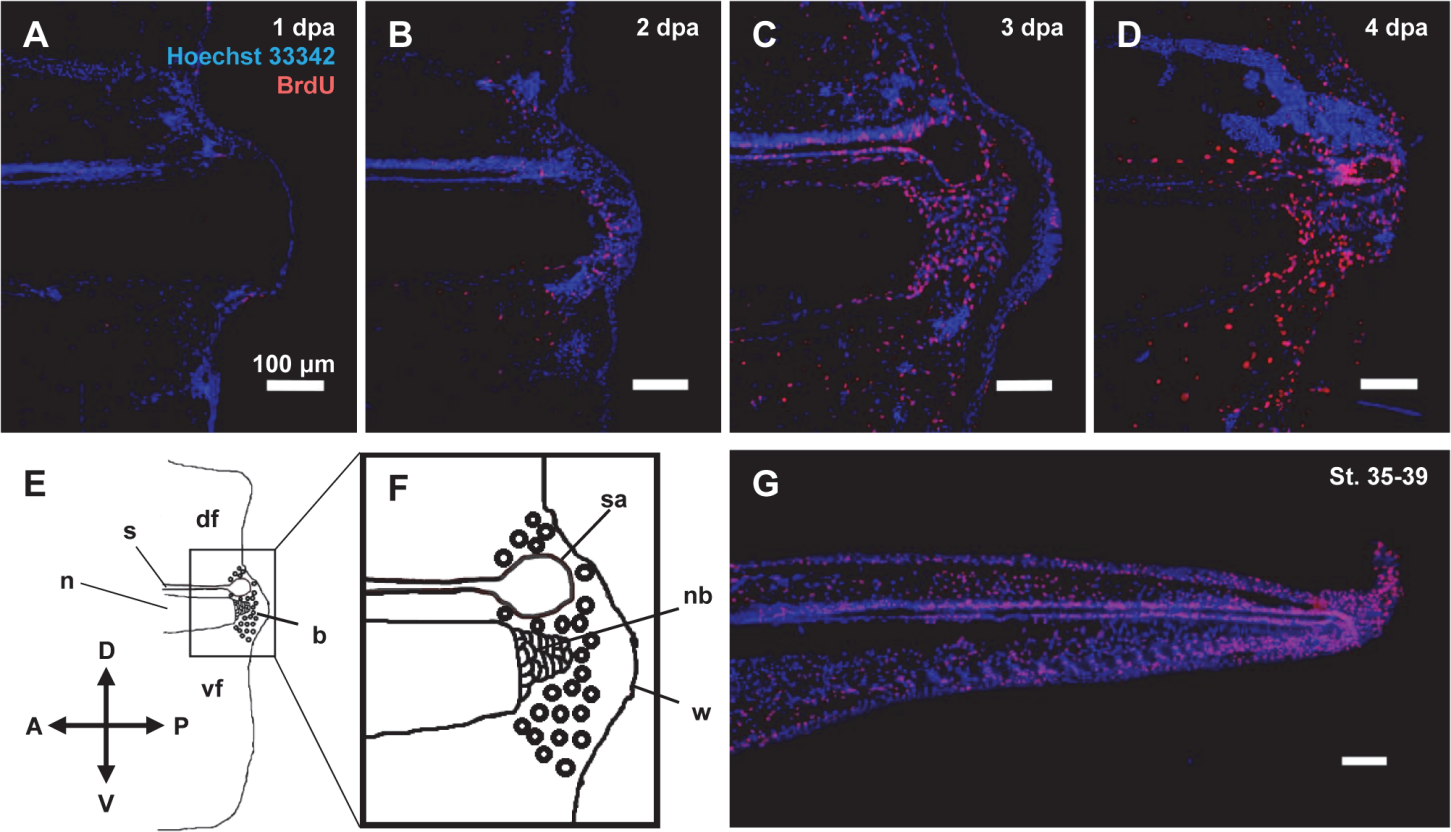
BrdU-immunohistochemistry of the proliferating cells in amputated tail stumps and tail buds. Sagittal sections from (A-D) tails stumps at 1, 2, 3, and 4 dpa of St. 49-53 tadpoles or (G) a tail bud of St. 35-39 tadpole were subjected to immunohistochemistry using anti-BrdU antibody (red), and the nuclei were counterstained with Hoechst 33342 (blue). BrdU-positive cells were enriched in the tail blastema at 3 dpa, and in the tail bud. (E) Schematic drawing of a 3-dpa tail stump. (F) A magnified view of the region delineated by the square in panel (E). Anterior is to the left, and dorsal is up. Scale bars indicate 100 μm. df, dorsal fin; s, spinal cord; n, notochord; b, blastema; vf, ventral fin; sa, spinal cord ampulla; nb, notochord bud; w, wound epithelium.

To isolate proliferating tadpole tail blastema cells, we first used BrdU immunohistochemistry to determine the period after tail amputation when the proliferating cells were most enriched in the tail blastema. We exposed tadpoles to water containing BrdU for 12 hours, and performed immunohistochemistry using anti-BrdU antibody and sections of tail stumps of tadpoles at 1, 2, 3, and 4 dpa (St. 49-53, the tadpoles were as a whole at the same stage at least morphologically over the time period). It revealed that the BrdU-positive cells were remarkably enriched in the tail blastema 3 dpa (Fig. 1A-D), so we collected the proliferating cells from 3-dpa blastemas. We also isolated proliferating tail bud cells from tail bud stage embryos as a control. Immunostaining using anti-BrdU antibody and sections of tail buds of the St. 35-39 tail bud stage embryos revealed many BrdU-positive cells in the tail bud (Fig. 1G).

**Fig 2:**
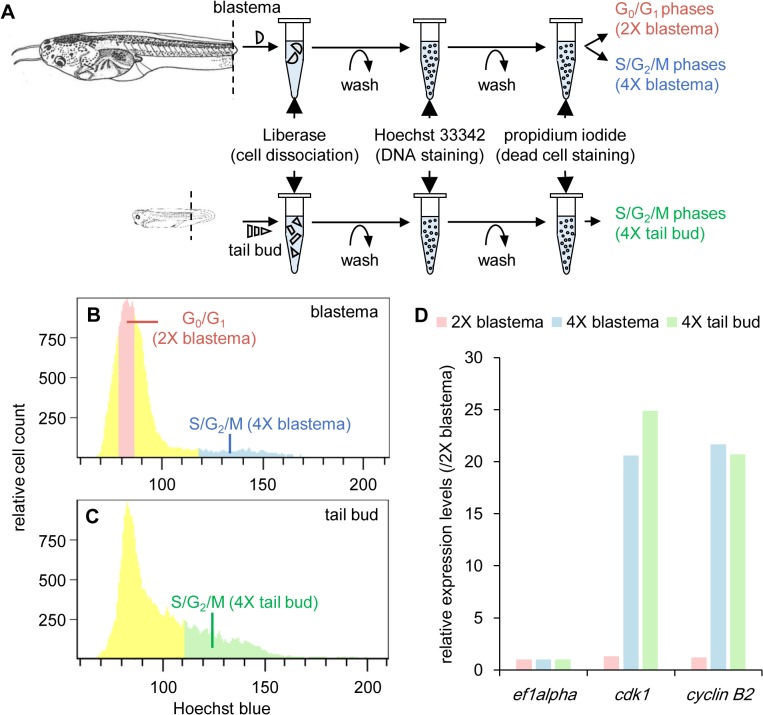
Isolation of proliferating and non-proliferating tail blastema cells and proliferating tail bud cells using flow cytometry. (A) A schematic drawing of the cell preparation procedure. *Xenopus* images were obtained from [18] and modified. (B,C) Cell cycle analysis of cells in (B) tail blastemas and (C) tail buds using flow cytometry. Horizontal axes represent relative fluorescent intensity of Hoechst blue, which reflects DNA contents, and vertical axes represent relative cell counts. The cells were classified into G_0_/G_1_ phases (major peaks), and S/G_2_/M phases (whose DNA contents were higher than those of cells in the major peak and lower than twice of those of cells in the major peak) based on the DNA content. Cell fractions colored in red (‘2X-tail blastema’ cell fraction), blue (‘4X-tail blastema’ cell fraction), and green (‘4X-tail bud’ cell fraction), respectively, were collected and subjected to RNA-sequence analysis. (D) Comparison of expression levels of cell cycle markers. Relative expression levels were estimated from RNA-sequencing data, taking the values in the ‘2X-tail blastema’ cell fraction as 1. Expression levels of *ef1α*, a housekeeping gene, were almost the same in all samples.

To isolate the proliferating tail blastema cells, we used flow cytometry to collect the cell fraction whose DNA content was 4X (S/G_2_/M phases). Single cell suspensions of the tail blastema and tail buds were prepared and stained with Hoechst 33342 for DNA staining, and propidium iodide for dead cell staining. When these cells were subjected to flow cytometry for cell cycle analysis, a major peak appeared that corresponded to G_0_/G_1_ phases as well as a cell population whose DNA contents were larger than those of the cells in the major peak and smaller than twice of those of the cells in the major peak, and seemed to correspond to S/G_2_/M phases, for both the tail blastema and tail bud (Fig. 2B, C). The ratio of cells in the S/G_2_/M phases was higher in the tail bud than in the tail blastema, consistent with the higher ratio of BrdU(+) cells detected in the tail bud than in the tail blastema (Fig. 1G). We isolated the cell fraction that corresponds to the S/G_2_/M phases (hereafter referred to as ‘4X-tail blastema’ cell fraction or R4 (regenerating 4X cell fraction)) for the tail blastema. We also isolated the cell fraction that corresponds to the G_0_/G_1_ phases (hereafter referred to as ‘2X-tail blastema’ cell fraction or R2) from the tail blastema sample as a control for the non-proliferating tail blastema cells, as well as the cell fraction that corresponds to the S/G_2_/M phases (hereafter referred to as ‘4X-tail bud’ cell fraction or E4 (embryonic 4X cell fraction)) from the tail bud sample as another control for the proliferating tail bud cells. As very large amounts of samples were needed for the RNA-sequencing analysis, flow cytometry was performed repeatedly and several batches of cell fractions were combined into single cell fractions. Fig. 2B and C are representative images of each flow cytometry experiment. During the cytometry experiment, fluorescent intensity of the cells slightly and continuously changed. Therefore, the cutoffs for the R4 and R2, and E4 and E2, could not be determined strictly throughout the experiments, and slightly varied depending on individual experiment. However, the later expression analysis of cell cycle marker genes indicated that this did not affected so much on the separation of the corresponding cell populations (see Fig. 2D).

### RNA-sequencing analysis of proliferating blastema cells, non-proliferating blastema cells, and proliferating tail bud cells

The RNAs extracted from these three cell fractions were then subjected to RNA-sequencing analysis. Approximately 80 million paired-end reads of length 100bp for each sample were sequenced (DRP002318). The sequenced reads were then mapped onto the *X*. *laevis* draft genome 7.0 [22–24]. Gene expression levels were estimated using Cufflinks [24]. The overall alignment rate was approximately 70%, and approximately 60% reads were aligned uniquely, which indicated that most reads were correctly mapped onto each alleles in the genome sequences, even though *X*. *laevis* is known as pseudotetraploid.

The gene expression levels of *cyclin-dependent kinase 1 (cdk1)* [28] and *cyclin B2* [29], both of which are cell cycle markers for G_2_/M phase cells in vertebrates, were approximately 20-folds higher in both R4 and E4 cell fractions than in the R2 cell fraction (Fig. 2D), confirming that the cells in the G_2_/M phases are enriched in the R4 and E4 cell fractions.

**Fig 3:**
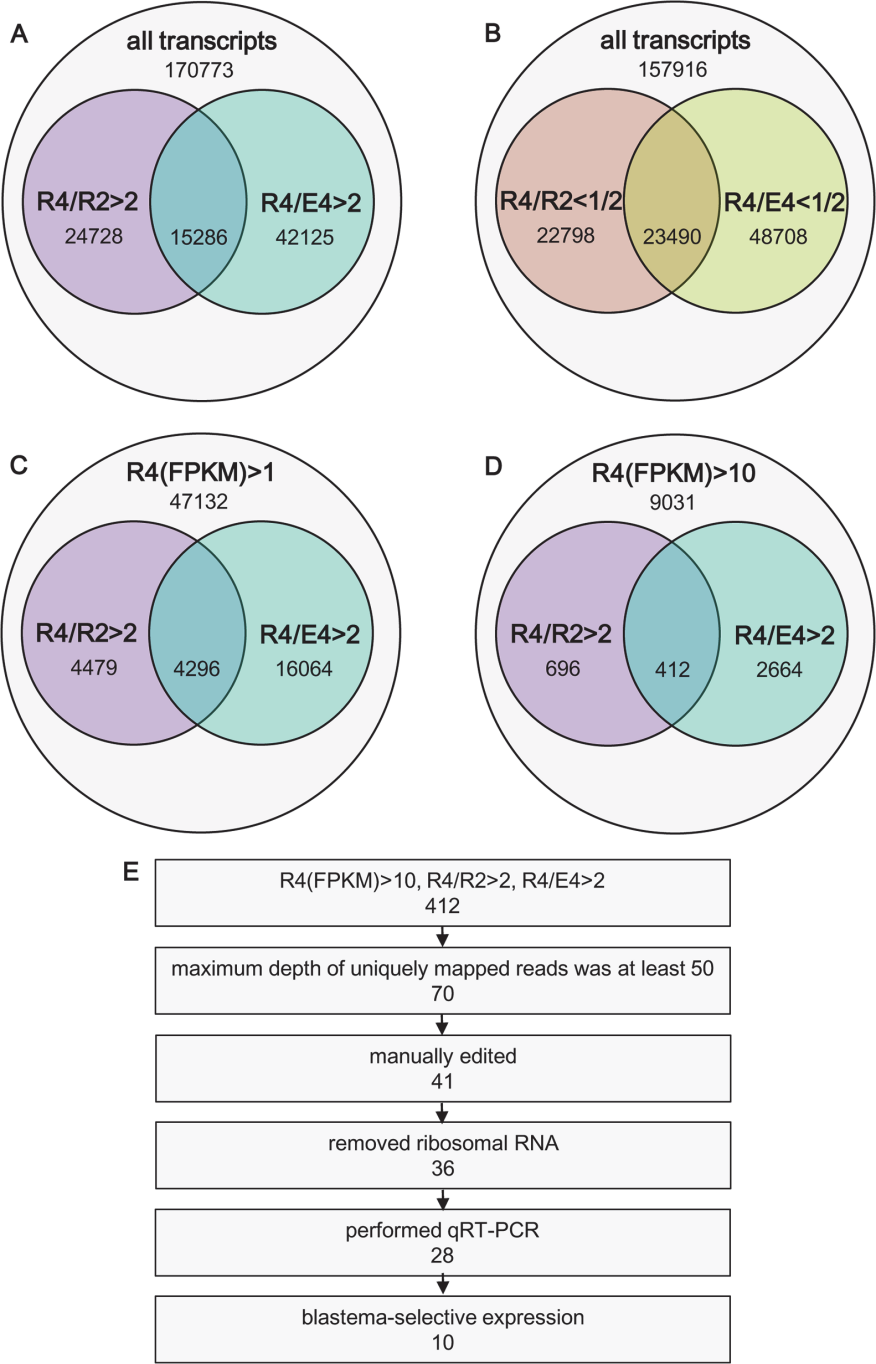
RNA-sequencing analysis of genes preferentially expressed in proliferating or non-proliferating tail blastema cells as well as proliferating tail bud cells. Genes preferentially expressed in each cell fraction were identified by RNA-sequencing analysis. (A) Genes that are up-regulated for more than 2-folds in R4 than in R2 or E4. (B) Genes down-regulated for more than 2-folds in R4 than in R2 or E4. (C,D) Genes whose FPKMs in R4 were larger than 1 (C) or 10 (D). (E) Flow chart for identification of genes expressed preferentially in the proliferating tail blastema cells.

The number of genes that were up-regulated for more than 2-fold in R4 than in R2 was smaller than that in R4 than in E4 (Fig. 3A). Similarly, the number of genes that were down-regulated for more than 2-fold in R4 than in R2 was smaller than that in R4 than in E4 (Fig. 3B). These observations suggest that the expression pattern between R4 and R2 is more similar than that between R4 and E4.

### Identification of genes expressed preferentially in proliferating tail blastema cells

As candidate genes expressed preferentially in the proliferating tail blastema cells, we identified the genes whose expression levels were more than two times higher in the R4 cell fraction than in the R2 and E4 cell fractions. In addition, to preferentially analyze highly expressed genes, we selected genes whose FPKMs (Fragments Per Kilobase of exon model per Million mapped fragments) for the R4 cell fraction exceeded 1, which corresponds to approximately 1/1000 of that of *ef1α* (Fig. 3C). However, considering the lack of replication of RNA-sequencing data, which limits the accuracy of the estimation of gene expressions, we decided to use more stringent cutoff, FPKM > 10 (Fig. 3D). As a result, 412 genes were listed as candidate genes. Assuming that estimations of FPKM from only a few mapped reads were inaccurate, we chose 70 genes, whose maximum depth of uniquely mapped reads was at least 50. We also excluded genes whose FPKM or gene models were considered to be inaccurate, and finally selected 41 genes. After exclusion of contaminated ribosomal RNA genes, a total of 36 genes were listed as candidates (Fig. 3E).

**Fig 4:**
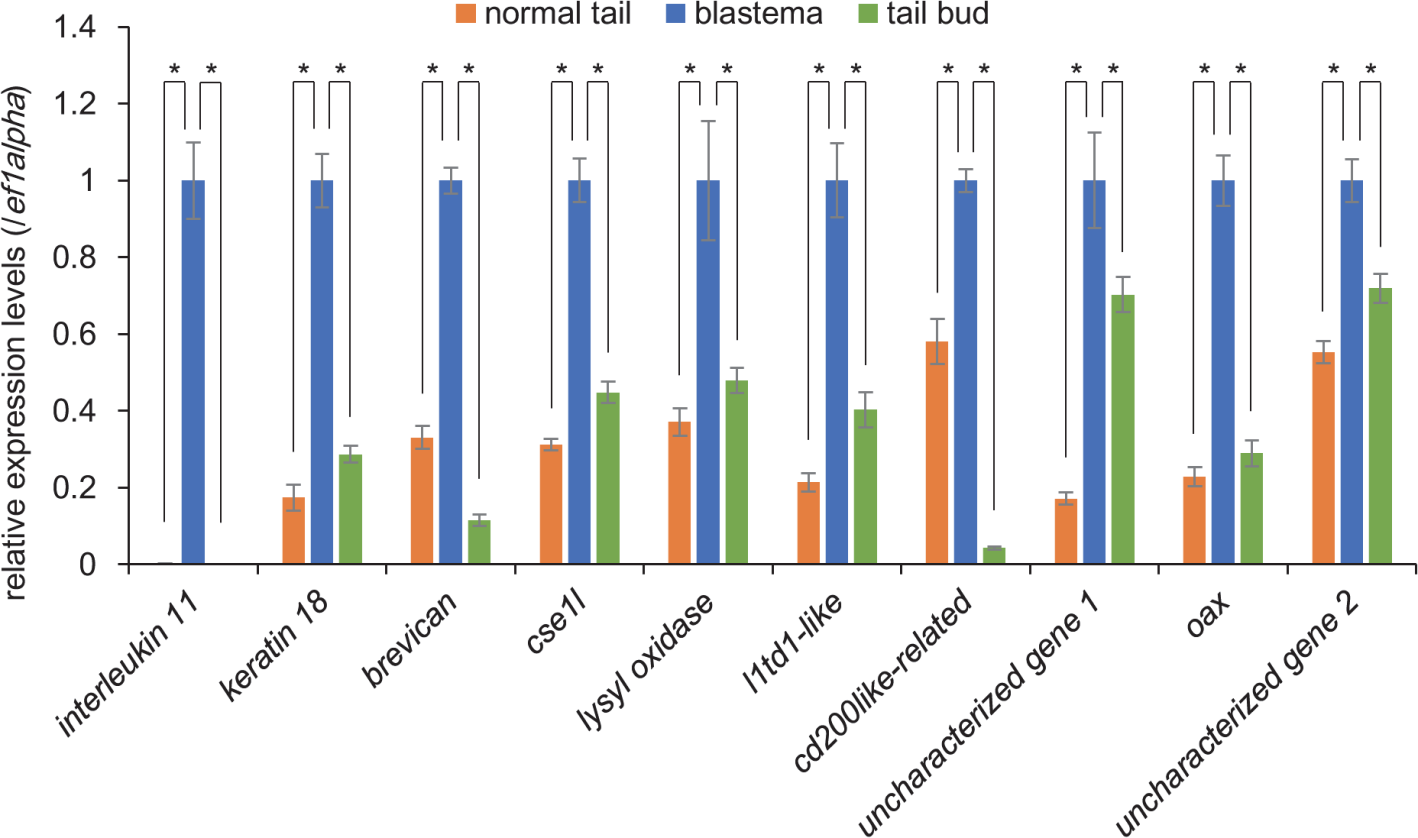
Identification of genes expressed preferentially in the tail blastema by qRT-PCR. The relative expression levels of the 10 genes which show the blastema-selective expression by qRT-PCR using RNAs extracted from normal tails (orange), regenerating tails (blue), and tail buds (green). The vertical axis represents relative expression levels calculated by taking the value of blastemas as 1, following normalization using those of *ef1α*. (mean ± SE, n = 4) *P < 0.05, Dunnett’s test. Abbreviations: *cse1l*, *chromosome segregation 1-like*; *l1td1*, *LINE-1 type transposase domain-containing protein 1*; *oax*, *oocyte activation in Xenopus*.

To confirm the reproducibility of differential gene expression of candidate genes identified by RNA-sequencing analysis, we performed qRT-PCR. As the expression levels of some candidate genes might have been perturbed during the cell dissociation and isolation procedures, we examined the expression levels of the candidate genes at the organ/tissue level using tail blastemas, normal tail tissues, and tail buds. Among the 36 candidate genes, we could design appropriate primers for qRT-PCR in 28 genes. 10 genes (*interleukin-11*, *keratin 18*, *brevican*, *cse1l*, *lysyl oxidase*, *l1td1-like*, *cd200like-related*, *oax*, and two uncharacterized genes) of the 28 genes were expressed at significantly higher levels in the tail blastemas than in the normal tail tissues and tail buds (Fig. 4).

We annotated these genes based on the homology search using blast [30] (S2 Table). The complete coding sequence (CDS) of *interleukin 11*, which showed most striking blastema-selective expression, were experimentally determined (AB933563). We also tried to identify longer sequences of the gene fragments of *l1td11-like*, *cd200like-related*, and uncharacterized gene 2, which were too short for further analysis, by 3’RACE and de novo assembly using the sequenced reads (DRP002318), and obtained the complete CDS of *cd200like-related* (AB933564), and the partial CDS of *l1td1-like* (AB933565). The gene models of these 10 genes were provided in S1 File.

**Fig 5:**
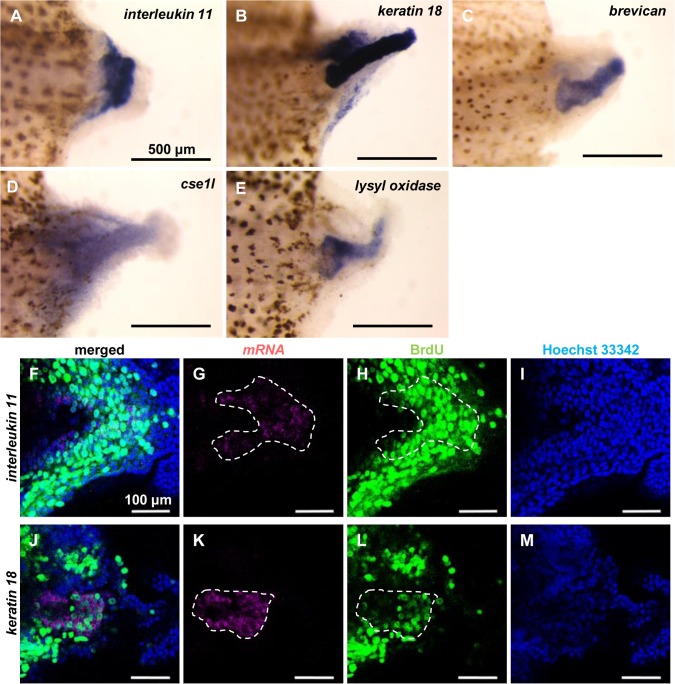
Expression analysis of candidate genes in regenerating tadpole tails by whole mount *in situ* hybridization. (A-E) Whole mount *in situ* hybridization using 3-dpa regenerating tails of St. 49-53 tadpoles for (A) *interleukin 11*, (B) *keratin 18*, (C) *brevican*, (D) *cse1l*, and (E) *lysyl oxidase* (blue/purple). (F-M) Sagittal sections of tail blastema from 3-dpa regenerating tails of St. 49-53 tadpoles double stained by whole mount *in situ* hybridization for (F-I) *interleukin 11* and (J-M) *keratin 18* (magenta) and BrdU-immunohistochemistry (green). (G) and (K) show magenta channels (mRNA), (H) and (L) show green channels (BrdU), (I) and (M) show blue channels (nuclei stained with Hoechst 33342), and (F) and (J) show merged images. Note that mRNA signals detected in the cytoplasm do not exactly merge with BrdU-signals detected in the nuclei. White broken lines indicate cell populations that highly expressed the genes. Anterior is to the left, and dorsal is up. Scale bars indicate 500 μm in (A-E) and 100 μm in (F-M).

We next used whole mount *in situ* hybridization to confirm that the candidate genes are actually expressed in the tail blastema. Among the 10 genes, we detected whole mount *in situ* hybridization signals for 5 genes, *interleukin-11*, *cse1l*, *keratin 18*, *brevican*, and *lysyl oxidase* (Fig. 5A-E). All five genes were expressed preferentially in the tail blastema, but their expression patterns in the tail blastema differed slightly. Both *interleukin-11* and *cse1l* were expressed in the broad region of the tail blastema, whereas *keratin 18*, *brevican*, and *lysyl oxidase* were expressed mainly in the notochord bud. Both *keratin 18* and *brevican* were also weakly expressed in the spinal cord ampulla, and *keratin 18* was also very weakly expressed in the round epithelium.

Finally, to more precisely identify the cell populations expressing these five genes in the proliferating tail blastema cells, we performed double-staining of BrdU-immunohistochemistry and whole mount *in situ* hybridization for the five genes. The whole mount *in situ* hybridization signals of *interleukin-11* and *keratin 18* were detected in restricted cell populations located at the inner regions of the BrdU-positive tail blastema cells (Fig. 5F-M).

## Discussion

In the present study, we analyzed gene expression profiles of *X*. *laevis* proliferating tail blastema cells, non-proliferating tail blastema cells, and proliferating tail bud cells.

Recently, Love *et al*. examined gene expression of the amputated stump of *X*. *tropicalis* tadpole tail using genome array, and reported that the expression of carbohydrate regulatory genes and genes relating to catalyzing reactive oxygen species change during regeneration [7,9,31]. When we searched the expression levels of those genes (S3 Table), however, expression of none of them changed drastically among three samples. In addition, *leptin* and *insulin* were almost not expressed, suggesting that the differences in experimental design or the materials or animals used results in these inconsistent results. In addition, we should be aware that the non-proliferating cell population may include some proliferating G_1_ cells, which might obscure the difference between the proliferating cells and non-proliferating cells.

We performed a comprehensive search for genes expressed preferentially in the proliferating tail blastema cells in *X*. *laevis* tadpoles to identify 10 genes, *interleukin-11*, *keratin 18*, *brevican*, *cse1l*, *lysyl oxidase*, *l1td1-like*, *cd200like-related*, *oax*, and two uncharacterized genes (Fig. 4). Among them, *interleukin-11* and *cse1l* were expressed in a broad area of the tail blastema, whereas *brevican*, *lysyl oxidase*, and *keratin 18* were mainly expressed in notochord buds (Fig. 5A-E). The finding that three of the five identified genes were expressed preferentially in the notochord bud likely reflects the relatively high ratio of notochord bud cells in the proliferating tail blastema cells (Fig. 1C).

Among the genes whose preferential expression in tail blastema was confirmed by qRT-PCR, *l1td1* is an RNA-binding protein required for self-renewal of human embryonic stem cells and proliferation of cancer cells [32]. It is thus possible that *l1td1-like* is required for self-renewal of proliferating tail blastema cells, even in *X*. *laevis* tadpoles.

*cd200* encodes an immunoglobulin superfamily membrane glycoprotein, which dampens immune system overactivation in mice [33]. A *cd200* isoform antagonizes *cd200* to inhibit its immunosuppressive action [34]. It is thus possible that *cd200like-related* plays a role in the modulation of autoimmune responses to tail blastema cells during the refractory period.

*oax* is a repetitive element transcribed by somatic nuclei when injected into the nuclei of *X*. *laevis* oocytes [35] and is thought to have originated by tandem duplication of a short interspersed repetitive element [36]. A transcript of a short interspersed repetitive element of mouse, B2 RNA, associates with RNA polymerase II to repress the transcription of certain genes during heat shock responses [37]. Interestingly, *oax* is expressed in the amputated stumps of regenerating *X*. *laevis* tadpole limbs [38]. Therefore, it is possible that *oax* plays a role in regulating gene expression in the tail blastemas, although the function of *oax* has not been clarified.

Among the five genes whose preferential expression in the tail blastema was confirmed by whole mount *in situ* hybridization, *cse1l* is highly expressed in proliferating cells [39], and its reduction leads to an accumulation of G_2_-arrested cells [40] in mammals. It is thus plausible that *cse1l* plays a role in regulating the cell cycle of proliferating tail blastema cells in the regenerating *X*. *laevis* tadpole tails.

Previous studies reported that *brevican* [41] and *lysyl oxidase* [42] are expressed in the notochord of the *X*. *laevis* embryos. *brevican* encodes a member of aggrecan-versican family proteoglycans that constitutes a component of the extracellular matrix [43]. *lysyl oxidase* catalyzes the covalent cross-link of the side chains of amino acid residues of collagen and elastin to stabilize the extracellular matrix [44]. *X*. *laevis* embryos treated with β–aminoproprionitrile, a specific inhibitor of the catalytic activity of lysyl oxidase family enzymes, develop kinks in the notochord [42]. It is thus highly likely that *brevican* and *lysyl oxidase* comprise the extracellular matrix, not only during tail development but also in the notochord bud during tail regeneration.

In the present study, we demonstrated that *interleukin-11* and *keratin 18* are actually expressed in a restricted cell population of proliferating tail blastema cells. A previous study reported that *keratin 18* is expressed in the regenerating newt limb blastema, and is necessary for cell proliferation [45]. Therefore, it is likely that *keratin 18* is also involved in the proliferation of notochord bud cells in the regenerating *X*. *laevis* tadpole tails. Interestingly, Ouro1 and Ouro2, which are members of the keratin superfamily, are expressed in the metamorphosing tadpole tails, and function as immune antigens, which leads to tail regression during metamorphosis [46]. It might be that *keratin 18* is recognized as an autoantigen by immature immune cells during the refractory period, which results in the impaired tail regenerative ability.

It is possible that *interleukin-11* expression in the proliferating tail blastema cells represents an immune response upon tail amputation. Viral [47] and bacterial [48] infection or stimulation by cytokines such as Interleukin-1, Tumor Necrosis Factor-α, and Transforming Growth Factor-β1 induce *interleukin-11* expression in mouse dendritic cells, macrophages, and other tissues [49]. Interleukin-11 stimulates megakaryocytopoiesis in human bone marrow mononuclear cells [50], which results in increased platelet production, and inhibits the production of proinflammatory cytokines from lipopolysaccharide-stimulated macrophages [51]. Thus, it might be that Interleukin-11 produced by the proliferating tail blastema cells facilitates wound healing and creates the appropriate conditions for successful tail regeneration in *X*. *laevis* tadpoles. On the other hand, a recent work revealed that *interleukin 11* is highly expressed in the injured heart of zebrafish, and its downstream signaling molecule, *janus kinase 1 (jak1)/ signal transducers and activators of transcription 3 (stat3)* is required for heart regeneration [52]. Therefore, regenerating tadpole tail may also need *jak1/stat3* signaling activated by *interleukin 11*. In addition, another study recently suggested that Interleukin-11 contributes to maintain the pluripotent state in induced pluripotent stem cells [53]. It is thus also plausible that *interleukin-11* contributes to maintain the pluripotent state of proliferating tail blastema cells in *X*. *laevis* tadpoles. A recent study reported that tissue-specific stem cells restricted to their cell lineages, but not dedifferentiated pluripotent stem cells, contribute to regenerate tissues in the *X*. *laevis* tadpole tail regeneration [54]. Expression of *interleukin-11* in a broad area of the tail blastema, except the wound epithelium, suggests that *interleukin-11* is involved in molecular mechanism(s) that are rather common among tissue-specific stem cells in the tail blastema of *X*. *laevis* tadpoles. Further analyses of the function of the genes identified in the present study will contribute to our understanding of not only the predicted autoreactive immune responses that impair tail regenerative ability during the refractory period, but also early molecular processes specific to organ/tissue regeneration.

## Supporting Information

S1 Table A list of primers used in qRT-PCR. (DOCX)

S2 Table Homology search for blastema selective genes. (DOCX)

S3 Table The expression of genes relating to carbohydrate metabolism or reactive oxygen species. (DOCX)

S1 File The gene models of the blastem selective genes. (ZIP)
