# Supplementary material for: Unique Gene Expression Profile of the Proliferating Xenopus Tadpole Tail Blastema Cells Deciphered by RNA-Sequencing Analysis
Source: PLoS One. 2015 Mar 16;10(3):e0111655. doi: 10.1371/journal.pone.0111655 (PMC4361676; doi:10.1371/journal.pone.0111655)
Supplement: S1 Table — (DOCX) [file pone.0111655.s001.docx]

| genes | forward primers | reverse primers |
| --- | --- | --- |
| *interleukin 11* | TCCTGAAGCTAAGCACTGACCT | TGAATTCCGTTAAATTCGTGGTCCA |
| *keratin 18* | CGCACCATGCAGTCCCTAGA | ACGTGCTTCTGTGTCGTGGA |
| *brevican* | CAAGTGGGGACGGTTCTGGT | GGTTGGGTTCCCTGAGTGCT |
| *cse1l* | TCGAGCTTCCCGAAGACGAC | AGCTGAGAGAAGGCTGCCTG |
| *lysyl oxidase* | CAACAGCAGCGTTTCCCAGG | GGTTGATGCTCGCAGTCTGC |
| *l1td1-like* | TTGTTGCCGTCCACCACTCG | AGAGGTCCGGAAGAGGCTCA |
| *cd200like-related* | CCACCTTCTGACTGTGTTGCA | TCAGGCAGAGAATGTATTTGCTCT |
| *uncharacterized gene 1* | GGTTCCACATTGGGCGTCAG | GCTAACCCAGCCTCTCAGCT |
| *oax* | AAAGCACTGCGGACAAGAGC | TAGCTCAGTCGGTAGAGCGC |
| *uncharacterized gene 2* | TGGCACAAGTGTGATGTTTCACG | CCCGAACTTTTGTTTCCGTTGTG |

**Supplemental Table 1 A list of primers used in qRT-PCR.**
