# Supplementary material for: Unique Gene Expression Profile of the Proliferating Xenopus Tadpole Tail Blastema Cells Deciphered by RNA-Sequencing Analysis
Source: PLoS One. 2015 Mar 16;10(3):e0111655. doi: 10.1371/journal.pone.0111655 (PMC4361676; doi:10.1371/journal.pone.0111655)
Supplement: S2 Table — (DOCX) [file pone.0111655.s002.docx]

**Supplemental Table2. Homology search for blastema selective genes.**

| clone | blastn(Xenopus) | blastx(all) | blastx(-Xenopus) | gene name |
| --- | --- | --- | --- | --- |
| A | no hit | turtle Interleukin 11 (XP_005312109.1) | - | *interleukin 11* |
| B | *X. laevis keratin 18* (NM_001088985.1) | - | - | *keratin 18* |
| C | *X. laevis brevican* (NM_001088637.1) | - | - | *brevican* |
| D | *X. laevis cse1l* (NM_001092566.1) | - | - | *cse1l* |
| E | *X. laevis lysyl oxidase* (BC130090.1) | - | - | *lysyl oxidase* |
| F | no hit | X. tropicalis L1td1-like (XP_004917530.1) | - | *l1td1-like* |
| G | *X. tropicalis uncharacterized mRNA* (XM_004918270.1) | X.tropicalis Uncharacterized protein (XP_004912498.1) | chinchilla CD200-like (XP_005386669.1) | *cd200like-related* |
| H | no hit | no hit | - | *uncharacterized gene 1* |
| I | reverse strand of *X. laevis oax* (AF225412.1) | - | - | *oax* |
| J | no hit | no hit | - | *uncharacterized gene 2* |

Top hits for blast search are listed. In blastn, top hits which matched query cover >0.3, and identity >0.7 are listed. blastn(Xenopus): blastn searching for Xenopus database, blastx(all): blastx searching for all non-redundant protein sequences, blastx(-Xenopus): blastx searching for all non-redundant protein sequences except Xenopus. turtle: *Chrysemys picta bellii*, chinchilla: *Chinchilla lanigera*.
