# Supplementary material for: Unique Gene Expression Profile of the Proliferating Xenopus Tadpole Tail Blastema Cells Deciphered by RNA-Sequencing Analysis
Source: PLoS One. 2015 Mar 16;10(3):e0111655. doi: 10.1371/journal.pone.0111655 (PMC4361676; doi:10.1371/journal.pone.0111655)
Supplement: S3 Table — (DOCX) [file pone.0111655.s003.docx]

**Supplemental Table3. The expression of genes relating to carbohydrate metabolism or reactive oxygen species.**

Abbreviations: pdgfa, platelet-derived growth factor alpha polypeptide; slc2a3, solute carrier family 2 member3; g6pd, glucose-6-phosphate dehydrogenase; cyba, cytochrome b-245 alpha polypeptide; hif1a, hypoxia-inducible factor 1, alpha subunit.

| Gene | FPKM(R2) | FPKM(R4) | FPKM(E4) |
| --- | --- | --- | --- |
| *leptin* | 0.5 | 0.6 | 0.0 |
| *insulin* | 0.0 | 0.0 | 0.0 |
| *pdgfa* | 8.3 | 14.2 | 4.4 |
| *slc2a3* | 2.8 | 2.8 | 2.9 |
| *g6pd* | 25.2 | 14.9 | 11.9 |
| *cyba* | 34.2 | 25.3 | 13.2 |
| *hif1a* | 26.4 | 30.6 | 22.0 |
